# The association of depression and perceived stress with beta cell function between African and Haitian Americans with and without type 2 diabetes

**DOI:** 10.4236/jdm.2013.34036

**Published:** 2013-11

**Authors:** Fatma G. Huffman, Maria Vallasciani, Joan A. Vaccaro, Joel C. Exebio, Gustavo G. Zarini, Ali Nayer, Sahar Ajabshir

**Affiliations:** 1 Department of Dietetics and Nutrition, Florida International University, Miami, USA; 2 Division of Nephrology and Hypertension, University of Miami, Miami, USA

**Keywords:** Beta Cell Function, HOMA-IR2, Type 2 Diabetes, Haitian Americans, African Americans, Depressive Symptoms, Perceived Stress

## Abstract

**Background:**

Diabetes and diabetes-related complications are major causes of morbidity and mortality in the United States. Depressive symptoms and perceived stress have been identified as possible risk factors for beta cell dysfunction and diabetes. The purpose of this study was to assess associations between depression symptoms and perceived stress with beta cell function between African and Haitian Americans with and without type 2 diabetes.

**Participants and Methods:**

Informed consent and data were available for 462 participants (231 African Americans and 231 Haitian Americans) for this cross-sectional study. A demographic questionnaire developed by the Primary Investigator was used to collect information regarding age, gender, smoking, and ethnicity. Diabetes status was determined by self-report and confirmed by fasting blood glucose. Anthropometrics (weight, and height and waist circumference) and vital signs (blood pressure) were taken. Blood samples were drawn after 8 – 10 hours over-night fasting to measure lipid panel, fasting plasma glucose and serum insulin concentrations. The homeostatic model assessment, version 2 (HOMA2) computer model was used to calculate beta cell function. Depression was assessed using the Beck Depression Inventory-II (BDI-II) and stress levels were assessed using the Perceived Stress Scale (PSS).

**Results:**

Moderate to severe depressive symptoms were more likely for persons with diabetes (p = 0.030). There were no differences in perceived stress between ethnicity and diabetes status (p = 0.283). General linear models for participants with and without type 2 diabetes using beta cell function as the dependent variable showed no association with depressive symptoms and perceived stress; however, Haitian Americans had significantly lower beta cell function than African Americans both with and without diabetes and adjusting for age, gender, waist circumference and smoking. Further research is needed to compare these risk factors in other race/ethnic groups.

## 1. INTRODUCTION

Diabetes and diabetes related complications are major causes of morbidity and mortality in the United States affecting approximately 25.8 million people, or 8.3% of the US population in 2010; these numbers are expected to rise by 2050 [1]. Approximately 43% of the population had some hyperglycemic condition and were at risk of developing diabetes [2]. As diabetes progresses, insulin sensitivity and beta cell function decline; albeit, the mechanisms of reductions are not fully understood [3]. Stress and depression may be risk factors for diabetes. There is evidence suggesting that beta cells have the ability to adapt to the hormonal changes produced by stressful events; however in the long term, psychological stress may induce pancreatic islet cell failure [4–6].

Insulin resistance is associated with hyperactivation of the hypothalamic-pituitary-adrenal (HPA) axis and excess plasma cortisol levels that may lead to depression [7]. Other studies supported these findings, and added that increased pro-inflammatory cytokines and disturbances of serotonin levels increased the risk of developing diabetes [8,9]. Individuals had a 37% higher risk of developing diabetes if they suffered from depressive symptoms [7,8,10]. Conversely, other studies encountered no association between hyperglycemia and insulin resistance with depression [9,11,12]. Further research is needed to clarify if there are any associations between markers of diabetes and depression.

The association of beta cell dysfunction with depression and perceived stress is controversial. African and Haitian Americans were selected for this study for several reasons. First, there is not much evidence of how perceived stress and depressive symptoms are associated with markers of diabetes in these populations. Second, most studies do not distinguish ethnic differences among Blacks. Yet, Blacks are high-risk groups for developing diabetes and diabetes complications [13]. Several studies have indicated that depression and perceived stress may be more pronounced in Blacks as compared to the general population [14]. Finally, due to the geographical location of South Florida, we have accessed Haitian and African Americans. Therefore, the primary aim of study is to assess if there is any association between depression symptoms and perceived stress with beta cell function between African and Haitians Americans with and without type 2 diabetes. The results could be important to increase awareness of depression and perceived stress symptoms in Blacks at risk for diabetes and diabetes-related complications.

## 2. PARTICIPANTS AND METHODS

### 2.1. Participants

This cross-sectional study was conducted between African and Haitian Americans with and without type 2 diabetes. The Institutional Review Board at Florida International University approved this study and initial data were collected 2008–2009. Written consent was obtained from the participants upon explaining the purpose and protocol of the study. For African Americans, recruitment was based on response to flyers sent from random selection of generated mailing lists that were purchased from Knowledge Base Marketing, Inc., Richardson, TX 75081. These mailing lists included African Americans with and without diabetes from Miami-Dade and Broward Counties, Florida. During a 1-year period, approximately 7550 letters were mailed to African Americans, with and without type 2 diabetes. Approximately 6% (n = 477) of the letters were returned due to unknown address. Response rate was 4% (n = 256).

There was no mailing list available for Haitian Americans. Haitian Americans (n = 259) were recruited from multiple community sources including local diabetes educators and community health practitioners in Miami-Dade and Broward Counties; Florida International University (FIU) faculty, staff and students; several residential rental facilities; and advertisements that were placed in local Haitian newspapers, churches, supermarkets and restaurants. In addition, radio advertisements on local Creole stations were aired. When the recruitment goal was achieved, recruitment efforts were stopped.

Participants were interviewed on the phone. The purpose of the study was explained and the gender and age of the subjects were assessed. In addition, age of diagnosis and treatment modality was obtained from those individuals who reported having diabetes. Eligible individuals’ participation was requested at the Human Nutrition Laboratory at Florida International University. Subjects participating in the study were asked not to smoke, consume any food or beverages except water, and not engage in any unusual physical activity for at least eight hours prior to their blood collection. Laboratory results revealed that twelve participants (African Americans = 4; Haitians = 8) who reported not having diabetes were reclassified as having type 2 diabetes in accordance to the American Diabetes Association standards (ADA). These participants were referred to their physicians.

### 2.2. Socio-Demographic Data

A standardized demographic questionnaire developed by the Primary Investigator was used to collect information regarding race/ethnicity, diabetes status, age, gender, smoking, and education level. Race/ethnicity was self-reported and confirmed by interview. Diabetes status was confirmed by laboratory results (see methods). Anthropometric measures were taken in the Nutrition Laboratory. Waist circumference was measured to the nearest 1 cm at a level midway between the lower rib margin and the iliac crest with a non-stretchable tape all around the body in a horizontal position. Weight measurements were taken using a SECA clinical scale and calibrating after each participant (SECA Corp, Columbia, MD). Height and weight were measured with a subject standing erect without shoes. BMI was calculated as weight (kg)/height (m^2^).

### 2.3. Blood Collection

Twenty milliliters (mL) of venous blood was collected from each participant after at least 8 hours of fasting by a certified phlebotomist using standard laboratory techniques. Two tubes were used to collect the blood samples: a tube containing ethylenediamine tetra-acetic acid (EDTA) to analyze hemoglobin A1c and a Vaccutainer Serum Separator Tube (SST) to analyze glucose. Glycated hemoglobin, reported as A1C percentages, was measured from whole blood samples, with the Roche Tina Quant method by Laboratory Corporation of America (LabCorp, FL, US). Glucose levels were measured by hexokinase enzymatic method after complete coagulated blood had been centrifuged at 2500 RPM for 30 minutes and placed into one labeled plastic tube. Serum insulin levels were assessed by Human Insulin ELISA kit from Millipore (St Charles, MZ, US).

### 2.4. Determination of Beta Cell Function

We calculated beta cell function using the homeostatic model assessment, version 2 (HOMA2) computer mode, which is based on the Oxford University HOMA2 calculator (www.ocdem.ox.ac.uk) [15]. It accounts for variations in peripheral and hepatic glucose resistance [16]. The new version, HOMA2 is designed to better match human physiology; it is an update of the HOMA1 based on modern insulin assays. The computer model, HOMA2, can be used to calculate beta cell function from radioimmunoassay insulin and paired fasting plasma glucose across a range of 1 – 2200 pmol/l for insulin, and 1 – 25 mmol/l for glucose [15]. For data analysis, subjects undergoing insulin treatment or have values <45 mg/dL or >450 mg/dL for plasma glucose and insulin <2.88 μIU/mL or >57.60 μIU/mL were excluded.

### 2.5. Psychosocial Factors

Depressive symptoms were assessed using the Beck Depression Inventory-II (BDI-II) [17]. This is a self-reported, 21-item questionnaire that measures the presence and severity of depressive symptoms using a 0 to 3 self-rating scale (0 being least depressed and 3 being most depressed). Summing scores for each question calculated a final BDI-II score. Moderate to severe symptoms of depression was defined as BDI-II score ≥16. A cutoff point ≥16 yielded a sensitivity of 0.73 and a specificity of 0.93 [18].

Stress levels were assessed using the Perceived Stress Scale (PSS) [19]. This is a 10-item scale that measures the subjects’ response to life stressors using a 4-point scale ranging from never (1) to always (4). The 10-item PSS has a higher psychometric quality than the 14-item version and it has been validated [19].

### 2.6. Statistical Analysis

Statistical analyses were performed using The Statistical Package for Social Sciences (SPSS), version 20. Data were presented as a mean ± standard deviation for general characteristics. A p value of <0.05 was considered significant. Prior to analyses, all continuous variables were tested for normality by the Kolmosorov-Simirnov test. Beta cell function was natural log transformed to achieve normality within diabetes status. Depressive symptoms, was converted into a binary variable based on a clinical cut off [9,20]. General linear models were used to test the relationships of depression and perceived stress with beta cell function, as the outcome.

Due to significant differences in beta cell function by diabetes status, separate models were run for each group (with and without diabetes). The clinically important covariates tested for the adjusted models were ethnicity, age, gender, ethnicity, waist circumference, education, and smoking status. A separate model for beta cell function in participants with diabetes was conducted with the adjustment covariates to which ‘years with diabetes’ was added. The same general linear models were run for perceived stress and depressive symptoms with insulin resistance as the dependent variable.

## 3. RESULTS

For this study, we had complete data for N = 462,234 without diabetes and 228 with diabetes (231 African Americans and 231 Haitian Americans). The general characteristics of the study population by ethnicity and diabetes status are shown in Table 1. Haitian Americans with diabetes as compared to the other groups, were the oldest (mean age 57.8 ± 10 years), had the lowest level of education and beta cell function, as well as the highest fasted plasma glucose, hemoglobin A1C and depression score. There were no differences in perceived stress among the groups. African Americans had a high rate of ever-smoking (approximately 40%) as compared to Haitian Americans (approximately 6%).

A difference of the means by ANOVA and post-hoc analysis showed a significant difference in depression score by ethnicity and diabetes status. Haitian Americans with diabetes scored higher (had more signs of depression) (mean = 10.9 ± 7.77) than Haitians and African Americans without diabetes (mean = 7.7 ± 7.85, p = 0.001); mean = 7.1 ± 7.50, respectively (p < 0.001). The difference in depression score between Haitian and African Americans with diabetes was not significant (p = 0.156).

The Chi-Squared test was used to assess depression across diabetes status. Moderate to severe depressive symptoms, an overall score of 16 or more, were more likely for the combined group of African Americans and Haitian Americans with diabetes, than without diabetes (p = 0.030). This relationships held for mild-moderate depressive symptoms or more (a score of 12 or more) (p = 0.029). There were no differences in perceived stress between ethnicity and diabetes status (p = 0.283). Adjusted general linear models with beta cell function as the dependent variable were run separately for participants with and without diabetes. Education was tested and not retained. The final model was adjusting for age, gender, waist circumference, and smoking. Depression and perceived stress were not associated with beta cell function for either model with (F_7,220_ = 3.14, p = −0.003) or without (F_7_,_226_ = 5.32, p < 0.001) diabetes. Haitian Americans with diabetes [b = −0.431 (−0.693, −0170) p = 0.001] and without diabetes [b = −0.151 (−0.277, −0.024) *p* = 0.020] had lower beta cell function as compared to African Americans. Years with diabetes, a factor in beta cell function, was not significantly correlated with ethnicity (Spearman’s Rho = −0.054, p = 0.428); however, we ran a general linear model with just those with diabetes entering years of having diabetes with the other adjustment variables with beta cell function as the dependent variable. The relationship with ethnicity and beta cell function remained. Haitian Americans had poorer beta cell function than African Americans [b= −0.391 (−0.685, −0.098), p = 0.009]. We tested insulin resistance as the dependent variable for the same models and found neither depressive symptoms, nor perceived stress was indicative of insulin metabolism (data not shown).

## 4. DISCUSSION

In the present study, we examined the associations between perceived stress/depressive symptoms and beta cell function in 462 African Americans and Haitian Americans with and without type 2 diabetes. To our knowledge, this is one of the few studies comparing beta cell function and psychosocial risk factors in two Black ethnicities with and without type 2 diabetes.

We found no significant association of depression with beta cell function, for Blacks with or without diabetes. Our results agreed with those of Lawlor *et al.* [21], which demonstrated no association between insulin resistance, as measured by HOMA levels, and depressive symptoms in a prospective study of middle aged men. In contrast, Pearson *et al.* [7] concluded that depressive disorder was significantly associated with insulin resistance as measured by HOMA independent of demographics, dietary and behavioral factors. It is important to note that the investigators measured depression disorder as opposed to depressive symptoms as measured by an index. There may be a bidirectional relationship between insulin metabolism and depression. Whether diabetes markers and its complications increase the risk of depression, or depression causes insulin resistance and consequently diabetes, is not known [9]. Moderate to severe depressive symptoms were positively associated with insulin resistance in males without diabetes for both a young cohort [9] and in older men [22].

In the present cross-sectional analysis, there was no relationship with perceived stress and beta cell function. It is important to note that risk factors for both stress and depression may overlap. Perceived overall stress caused by exposure to unfavorable environmental and lifestyle factors was positively related to insulin resistance, obesity, decreased adherence to treatment and medications, and therefore, poor diabetes outcomes [11,23,24]. Thomas *et al.* [10] suggested that some individuals were more susceptible to depression due to genetic as well as socioeconomic factors. In addition, racial discrimination has a negative impact on psychological well-being. The secretion of glucocorticoids during periods of stress causes hyperglycemia and it is associated with insulin resistance [25]. Novak *et al.* [26] found that men who reported permanent stress during many years had an increased risk of developing type 2 diabetes, independent of demographic, socioeconomic, clinical, and lifestyle factors. Conversely, perceived stress was associated with beta cell dysfunction in several studies [27,28]; however, stress was measured with physiological indicators as opposed to reported perceived stress.

Stress may be more pronounced for minorities and economically disadvantaged individuals [14,29]. Living in a disadvantaged environment with limited access to health care produces a stressful environment and increases risk of depression and diabetes for African Americans living in poverty [14,29]. Several unhealthy behaviors used for coping with stress have been suggested that lead to negative health outcomes among Blacks [14]. Krishnan *et al.* [30] suggested that chronic stress may lead to insulin resistance and diabetes among Backs. In addition, perception of racism was related to increased levels of stress and poor diabetic outcomes. Wagner *et al.* [31] found that African Americans linked racism to decreased diabetes self-management and control through negative emotions, physiological arousal, and inadequate coping mechanisms.

Earlier studies, using HOMA1 were in agreement with our findings for obesity and insulin metabolism [32,33]. In our study, we found a positive relationship between waist circumference and insulin resistance and a negative relationship between waist circumference and beta cell function as measured by HOMA2. Chung *et al.* [5] reported that obesity measured by body mass index was negatively associated with beta cell function and positively associated with insulin resistance as measured by HOMA1. Everson-Rose *et al.* [33] reported that associations between depressive symptoms and insulin resistance were mediated through central adiposity and obesity, and that the relationship was significant among African American women. Although African American participants had higher beta cell function that Haitian Americans regardless of diabetes status, they also had a higher percent of obesity, as compared to Haitian Americans. Beta cell dysfunction may be a result of loss of beta cell mass, which contributes to hyperglycemia of type 2 diabetes, but is not clearly understood on a genetic and molecular level [3]. There is evidence stating that in order to maintain normal levels of blood glucose, African Americans have a compensatory increase in beta cell function; however, progressive loss of the ability to compensate for a decreased in insulin sensitivity increases the risk of type 2 diabetes [34].

The results of this study are in accordance with the literature finding higher depression in persons with diabetes. Both African and Haitian Americans with diabetes were more likely to have moderate to severe depression than those without diabetes. Thomas *et al.* [10] reported that the diagnosis of type 2 diabetes and its complications was associated with increased depression disorder independent of education and gender in a group of low-income adults. Several studies explained that unfavorable environmental and lifestyle factors, in combination with diabetes and its complications, could lead to depression in the general population. There are several possible explanations as to why environmental factors, together with diabetes, may increase the risk of depression. Depression is mainly caused by a disruption of social, psychological, behavioral, and biological factors [8]. Studies reported that poor eating habits, physical inactivity, low socioeconomic status and education level, smoking, and limited access to health care services and healthy foods were risk factors for depression [7,8,11, 29,35–37].

We found Haitian Americans with diabetes to be more likely to have moderate to severe depressive symptoms than Haitian or African Americans without diabetes. Haitian American participants had a lower education level as compared to African Americans and this may be a contributing factor. Higher levels of depression were associated with limited economic and social resources in a cohort of African American women at risk for diabetes [29]. Many Black Americans live in precarious and stressful environments, and engaged in many unhealthy behaviors to be able to cope with stress and depression [14]. Therefore, overall stressful life events may outweigh diabetes-related stress and depression. We found no difference in perceived stress between the Black ethnicities or by diabetes status. Moreover, in our study of self-rated health in this cohort, we found perceived stress was higher for individuals who were younger, female, less educated, and consumed higher calories, regardless of ethnicity or diabetes status [37].

This study has several limitations. First, this was not a population-based study and recruitment techniques differed between African and Haitian Americans. Second the cross-sectional nature of the study limits the establishment of causal relationships between variables. Third, depressive symptoms and perceived stress were self-reported, increasing subjectivity of the responses. Finally, although we included education, we did not measure or account for socioeconomic status, which may increase depressive symptoms and perceived stress in African and Haitian Americans. Despite these limitations, this study is one of the few providing data on the Black American population, differentiating Haitian and African Americans.

## 5. CONCLUSION

In conclusion, Haitian Americans with type 2 diabetes were more likely to have severe depressive symptoms (BDS ≥ 16) than African and Haitian Americans without type 2 diabetes. Despite having smaller waist circumferences, Haitian Americans had worse beta cell function than African Americans. These results suggest that early screening for insulin metabolism and depression may improve medical care of Blacks at risk for diabetes.

## Figures and Tables

**Table 1.** General characteristics of the study participants by ethnicity and diabetes.

Variable	AA with T2D	AA without T2D	HA with T2D	HA without T2D	P

Age (years)	54.4 ± 10.1	50.7 ± 8.5	57.8 ± 10.0	54.2 ± 11.0	<0.001
FPG (mg/dL)	147.3 ± 66.0	95.8 ± 12.9	162.6 ± 84.5	99.5 ± 16.6	<0.001
Hemoglobin A1c (%)	7.6 ± 1.9	5.9 ± 0.4	8.5 ± 2.6	5.9 ± 0.5	<0.001
Insulin (pmol/L)	16.3 ± 15.8	12.2 ± 8.9	8.9 ± 8.7	9.9 ± 5.6	<0.001
Beta cell function (HOMA2)	63.5 ± 53.9	108.5 ± 47.4	42.2 ± 46.2	93.3 ± 42.9	<0.001
Insulin resistance (HOMA2)	2.1 ± 1.5	1.6 ± 1.2	1.4 ± 1.0	1.3 ± 0.7	<0.001
Depression score^*^ (0 – 63)	9.5 ± 9.7	7.1 ± 7.5	10.9 ± 7.8	7.7 ± 7.8	<0.001
Perceived stress (0 – 40)	20.4 ± 5.8	20.2 ± 5.7	20.1 ± 5.7	19.1 ± 6.2	0.282
Waist circumference (cm)	114.6 ± 17.5	102.1 ± 14.5	99.2 ± 11.4	96.2 ± 12.5	<0.001
Gender	-	-	-	-	0.841
Female	67 (52.3)	59 (50.0)	76 (55.1)	60 (50.4)	
Male	61 (47.7)	59 (50.0)	62 (44.9)	59 (49.6)	
Smoked >100 cigarettes (yes)	47 (36.7)	48 (40.7)	9 (6.5)	7(5.9)	<0.001
Education	-	-	-	-	<0.001
<High school	22 (17.2)	16 (13.6)	74 (53.6)	48 (40.3)	
High school	41 (32.0)	37 (31.4))	29 (21.0)	25 (25.0)	
Some college	48 (37.5)	42 (35.6)	19 (13.8)	23 (19.3)	
College or more	17 (13.3)	23 (19.5)	16 (11.6)	23 (19.3)	
Moderate to severe depressive symptoms (≥16)	23 (18.0)	15 (12.7)	23 (16.7)	12 (10.1)	0.268

AA: African Americans; HA: Haitian Americans; T2D: type 2 diabetes mellitus; FPG: fasting plasma glucose; HOMA2: homeostatic model assessment, version 2. Continuous variables are given as means ± SD and categorical variable are shown as number (%).

* Depression score measures depressive symptoms.
